# The use of 2D fingerprint methods to support the assessment of structural similarity in orphan drug legislation

**DOI:** 10.1186/1758-2946-6-5

**Published:** 2014-02-01

**Authors:** Pedro Franco, Nuria Porta, John D Holliday, Peter Willett

**Affiliations:** 1 European Medicines Agency, 7 Westferry Circus, Canary Wharf, London E14 4HB, UK; 2 Information School, University of Sheffield, 211 Portobello Street, Sheffield S1 4DP, UK

**Keywords:** Drug registration, European Medicines Agency, Fingerprint, Molecular similarity, Ophan drug, Similarity

## Abstract

**Background:**

In the European Union, medicines are authorised for some rare disease only if they are judged to be dissimilar to authorised orphan drugs for that disease. This paper describes the use of 2D fingerprints to show the extent of the relationship between computed levels of structural similarity for pairs of molecules and expert judgments of the similarities of those pairs. The resulting relationship can be used to provide input to the assessment of new active compounds for which orphan drug authorisation is being sought.

**Results:**

143 experts provided judgments of the similarity or dissimilarity of 100 pairs of drug-like molecules from the DrugBank 3.0 database. The similarities of these pairs were also computed using BCI, Daylight, ECFC4, ECFP4, MDL and Unity 2D fingerprints. Logistic regression analyses demonstrated a strong relationship between the human and computed similarity assessments, with the resulting regression models having significant predictive power in experiments using data from submissions of orphan drug medicines to the European Medicines Agency. The BCI fingerprints performed best overall on the DrugBank dataset while the BCI, Daylight, ECFP4 and Unity fingerprints performed comparably on the European Medicines Agency dataset.

**Conclusions:**

Measures of structural similarity based on 2D fingerprints can provide a useful source of information for the assessment of orphan drug status by regulatory authorities.

## Background

The discovery, testing and registration of a novel drug is both time-consuming and extremely expensive, with a review by Morgan *et al*. quoting costs in the range $161 million to $1.8 billion for the development of a novel therapeutic agent [1]. Such huge costs are acceptable to a pharmaceutical company if, and only if, there is a reasonable expectation that they can be recouped and a profit achieved when the drug is made available to large numbers of patients suffering from the target disease. There are, however, many diseases where there is a clear need for treatment but where there are insufficient patients world-wide to support the costs of modern drug research. These medical conditions are normally referred to as *rare diseases* and there is much current interest in the development of *orphan drugs* for the treatment of such diseases [2-4].

There is no single definition of a rare disease, since account may need to be taken not only of the number of patients affected by it but also its severity and the availability of existing, adequate treatments. Different regulatory authorities have hence adopted rather different definitions [5-7]. In the European Union (EU), which is the context for this paper, the evaluation of orphan drugs is coordinated by the European Medicines Agency (hereafter the EMA). According to article 3 (1) of *Regulation (EC) No 141/2000 of the European Parliament and of the Council of 16 December 1999 on orphan medicinal products*, a medicine must meet a number of criteria if it is to qualify as an orphan drug: “it must be intended for the treatment, prevention or diagnosis of a disease that is life-threatening or chronically debilitating; the prevalence of the condition in the EU must not be more than 5 in 10,000 or it must be unlikely that marketing of the medicine would generate sufficient returns to justify the investment needed for its development; and no satisfactory method of diagnosis, prevention or treatment of the condition concerned can be authorised, or, if such a method exists, the medicine must be of significant benefit to those affected by the condition”.

The EU provides a range of incentives to encourage the development of orphan drugs, the most important of which is a high level of market exclusivity: once a medicine has been awarded an orphan drug authorisation by the European Commission, no similar medicinal product can be brought to the European market for a period of ten years. The criteria and incentives were detailed formally, in the regulation noted above, but without any explicit specification of the nature or the extent of the similarity required to define a “similar medicinal product”. This lack was addressed, in part at least, in a subsequent regulation - *Commission Regulation (EC) No 847/2000 of 27 April 2000 laying down the provisions for implementation of the criteria for designation of a medicinal product as an orphan medicinal product and definitions of the concepts ‘similar medicinal product’ and ‘clinical superiority’ -* which defined a similar active substance as “an identical active substance, or an active substance with the same principal molecular structural features (but not necessarily all of the same molecular structural features) and which acts via the same mechanism”.

When a company applies to register a new medicine for an indication that has already been granted for an orphan medicine it is the responsibility of the EMA’s Committee for Medicinal Products for Human Use (CHMP) to decide if the new drug is indeed similar to an existing orphan drug, with an application being successful only when the CHMP decides that this is not the case. To date, the evaluations carried out by the CHMP have been based largely on human judgments of similarity. In this paper, we discuss the use of computed measures of structural similarity based on 2D fingerprints to provide an additional source of information that could be used when the CHMP considers the relationships that may exist between existing and proposed new medicines for rare diseases.

## Results and discussion

### Human similarity judgements

**Figure 1:**
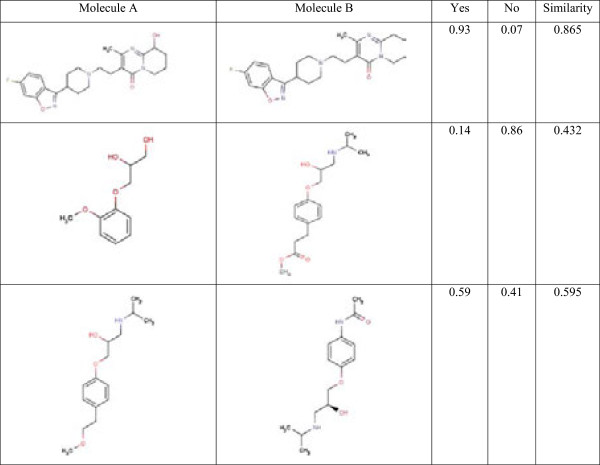
**Three training-set molecule-pairs with the corresponding fractions (*n*** **= 143) of Yes/No responses to the question “Are these molecules similar?” The similarity values in the right-hand column are those obtained using the Tanimoto coefficient and ECFP4 fingerprints.**

The 143 experts provided Yes/No decisions on the training-set of 100 DrugBank 3.0 molecule-pairs as detailed in Additional file 1: Table S1 (see Experimental methods). Figure 1 shows three typical pairs, the corresponding proportions of Yes/No responses to the question “Are these molecules similar?”, and the Tanimoto similarity computed using ECFP4 fingerprints.

**Figure 2:**
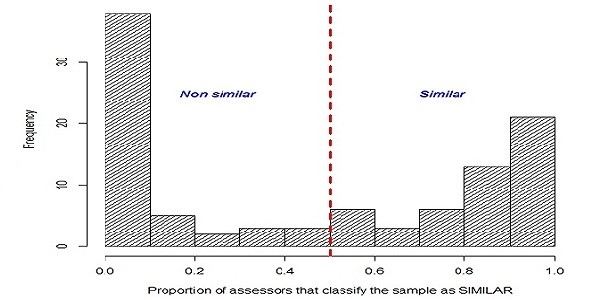
Distribution of expert assessments on the training-set.

The distribution of the similarity judgments provided by the experts for each of the molecule-pairs is shown in Figure 2. The left-hand column of the plot shows, for example, that there were 38 molecule-pairs where < 0.1 of the judgments were that the molecules were similar (i.e., the great majority of the experts considered these to be non-similar molecule-pairs); the right-hand column, conversely, shows that there were 21 molecule-pairs where ≥0.9 of the judgments were that the molecules were similar (i.e., the great majority of the experts considered these to be similar molecule-pairs). If the experts had been in total agreement with each other then the plot would simply have consisted of these two columns, without any of the intervening columns, which denote molecule-pairs where there was some level of disagreement. However, inspection of the plot shows that there were 41 molecule-pairs with fractions in the range 0.1-0.9, including nine where very considerable levels of disagreement were evident, *viz* the two middle columns representing the molecule-pairs where 0.4-0.6 of the experts felt that the two molecules were similar.

We ascribe these levels of disagreement to the inherently subjective nature of similarity [8,9], with an individual’s perception that two objects are similar depending on a range of factors (such as their state of mind, gender, age, personality and previous scientific experience *inter alia*). That being so, it is hardly surprising that different experts responded in different ways to the molecule-pairs that were presented to them, a finding that is consistent with previous experimental studies that have demonstrated that different individuals do not perceive chemical structure information in the same way. For example, Lajiness *et al*. report a study of medicinal chemists at Pharmacia, who were asked to review lists of compounds in order to assess their potential as leads in a drug discovery programme [10]; not only were there marked inconsistencies between the chemists, but even the same chemist might give different assessments on different occasions. Hack *et al*. reported an analogous study that sought to enhance the diversity of the Johnson & Johnson corporate structure database; they found that whilst there were considerable differences between individual chemists a fair level of consistency could be achieved using a wisdom-of-crowds approach [11]. This technique was also used by Oprea *et al*. to reconcile the often disparate views of pharmaceutical experts as to the effectiveness of chemical probes resulting from the NIH Molecular Libraries and Imaging Initiative [12]. Boda *et al*. [13] and Bonnet [14] studied groups of medicinal chemists’ assessments of molecular synthetic feasibility, and again observed some degree of inconsistency in the judgments that were made. Finally, Kutchukian *et al*. have reported a large-scale study of medicinal chemists at Novartis, who were asked to select chemical fragments for lead-generation projects. There was not only a marked level of inconsistency in the selection, but also a comparable level of inconsistency in the reasons for their selections [9]. Analogous variations in the ways that individuals react to objects have been widely observed: for example, when indexing terms are assigned to documents [15], when links are created in hypertext systems [16], when search strategies are chosen for accessing text databases [17], and when scientists create mental maps of active research areas [18].

We note here one characteristic of the training data that could have affected the results, which is the way that the molecules were presented to the experts for assessment. In some cases, the molecules in a pair were displayed in such a way that the structural similarities were obvious to the human eye with the common features clearly aligned, as exemplified by the molecules in the first row of Figure 1. In other cases, the similarities may have been less obvious when the common features were not aligned, as exemplified by the molecules in the third row of Figure 1. Such variant alignments could result in the experts perceiving the molecules comprising a pair to be less similar than might have been expected from one or more of the computed, fingerprint-based similarities. The alignments presented to the experts (such as the examples above and the molecule-pairs in Additional file 1: Table S1) were those available in the DrugBank database. No attempt was made to modify the alignments in cases where it was felt that improvements were possible (such as the example above), since this is the situation faced by the members of the CHMP when they consider applications for authorisation; indeed, they have the additional problem that the molecule-pairs that they inspect (i.e., a molecule that has been submitted for consideration and the existing orphan-drug for that disease) may well have been drawn using different drawing packages.

### Logistic regression, receiver operating characteristic curves and performance statistics

Once the human judgments had been determined and the similar and non-similar molecular-pairs identified, it was possible to develop logistic regression models that assessed how fingerprint-based similarities were correlated with the probability of being considered similar by the majority of the experts. For each fitted model, ROC curves and other performance statistics were computed in order to assess the predictive performance of each fingerprint. The analysis is exemplified by the logistic regression model for the ECFP4 fingerprint, with the same processes being applied to each of the fingerprints.

**Figure 3:**
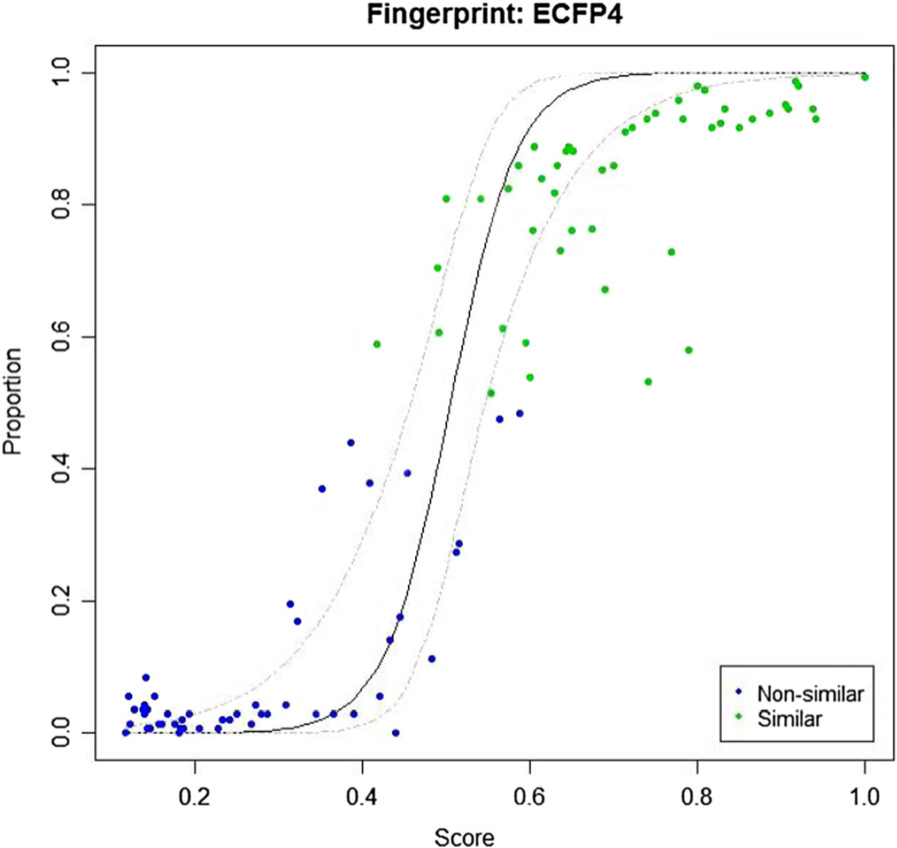
**Plot of the proportion of experts who assessed a training-set molecule-pair as being similar against the ECFP4 similarity for that molecule-pair.** The figure also shows the computed logistic regression curve (and 95% confidence limits) for this fingerprint.

**Figure 4:**
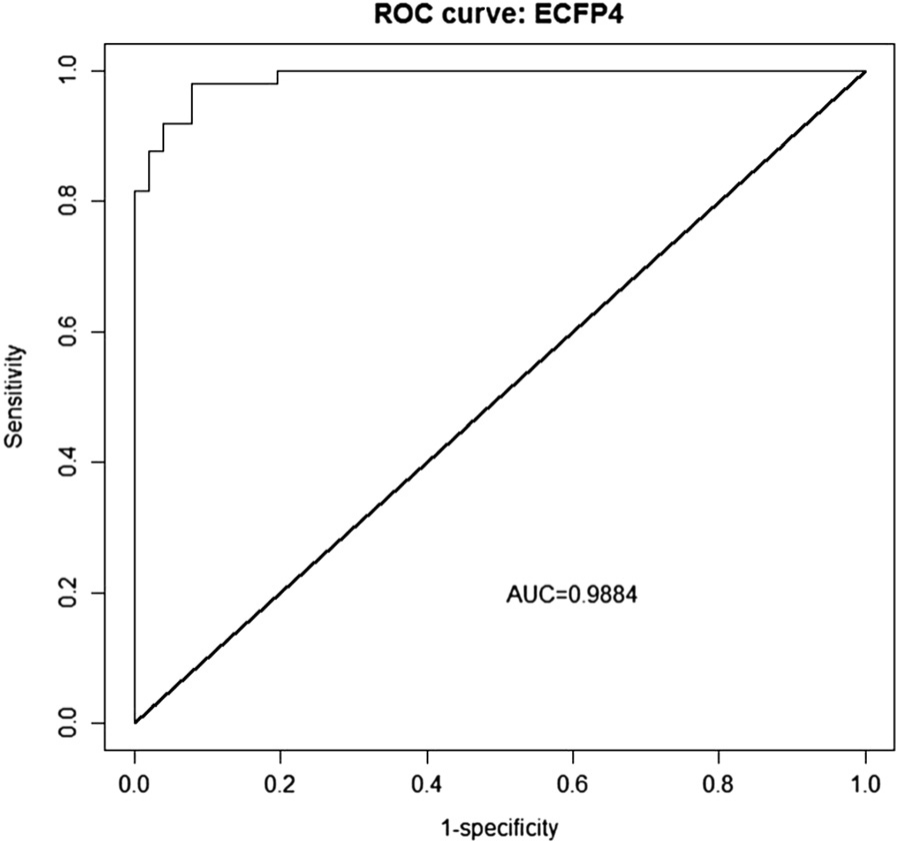
ROC curve for ECFP4 fingerprints.

Figure 3 plots the proportion of the expert assessors who judged a molecule-pair as being similar (Y-axis) against the computed ECFP4 similarity score for that molecule-pair (X-axis). It will be seen that there is an excellent separation of similar and non-similar molecular pairs (in green and blue, respectively), with smaller ECFP4 values corresponding to molecules considered not similar, and greater ECFP4 values corresponding to those considered similar. In addition to the observed data, the solid line in Figure 3 represents the estimated probability of being similar as predicted by the logistic regression model (together with the 95% confidence limits for this prediction). Table 1 contains the estimates of β_0_ and β_1_ for the ECFP4 regression model (see Experimental methods for the definition of these parameters), which are −12.754 and 2.524, respectively. The latter value means that for each increment of 0.1 in the ECFP4 score, the odds of a molecule-pair being classified as similar are multiplied by exp(2.524), i.e., 12.48 times. The Nagelkerke *R*^2^ value is high (0.894), indicating a good fit of the model to the data. The computed value for the threshold similarity (i.e., the ECFP4 value for which the corresponding probability of being classified as similar by the experts is 0.5), *t*_*LR*_, is 0.505. The ECFP4 ROC curve is shown in Figure 4, where it will be seen that a very high AUC value (0.988) is obtained, indicating the ability of this fingerprint to discriminate between similar and non-similar molecule-pairs.

**Table 1:** Logistic regression to predict the similarity, or otherwise, of training-set molecule-pairs using different types of fingerprint

**Fingerprint**	**β**_**0**_	**β**_**1**_	***R***^**2**^	***t***_***LR***_	**AUC**
BCI	−12.758	2.128	0.906	0.599	0.990
Daylight	−10.677	1.850	0.884	0.577	0.986
ECFC4	−9.207	2.438	0.878	0.378	0.983
ECFP4	−12.754	2.524	0.894	0.505	0.988
MDL	−9.022	1.380	0.812	0.654	0.973
Unity	−12.347	1.956	0.884	0.631	0.987

The columns contain the β_0_ and β_1_ values for the logistic regression model, the Nagelkerke *R*^2^ value, the computed value for *t*_*LR*_ and the AUC for the ROC curve.

The corresponding results for all of the six fingerprints are listed in Table 1, with the fingerprints listed in alphabetical order. As with the ECFP4 fingerprint, goodness-of-fit assessment was performed for all fingerprints and the assumptions of each fitted model were assessed with the Hosmer-Lemenshow test. The Nagelkerke *R*^2^ values were high (>0.8) for all fingerprints. In the case of the Unity data, the model listed in Table 1 is that obtained after the elimination of one outlier molecule-pair (number 99 in Additional file 1: Table S1), where over 70% of the experts judged the pair to be similar despite a Unity similarity of just 0.217. When this molecule pair was included in the model, the assumptions of the logistic regression did not hold: removing this observation, nevertheless, did not change significantly the estimates of β_0_ and β_1_.

As discussed below (see Experimental methods), the various performance statistics were computed as the value of *t,* the threshold similarity, was systematically varied, so as to determine the cut-off value, *t*_*ROC*_, that resulted in the best overall predictive performance. The results of these experiments are shown in Table 2, in the column headed Probability, which lists the probability that a molecule-pair will be judged as similar if the similarity fingerprint value is at least the specified value of *t*_*ROC*_. It will be seen that the best overall performance would appear to come from use of the BCI fingerprints with a similarity threshold of 0.606, this giving the largest observed values for the accuracy, the *F* index, the Youden index and the Matthews coefficient, and the second largest observed value for the precision. The *t*_*ROC*_ values in this table are very close to the corresponding *t*_*LR*_ values in Table 1, with the sole exception of the Daylight fingerprints (0.510 and 0.577, respectively). This was expected as the prevalence of similar molecules in the training-set was close to 50%, the probability cut-off used to compute *t*_*LR*_.

**Table 2:** Optimal levels of performance using ROC curves

**Fingerprint**	***t***_***ROC***_	**Probability**	**Sensitivity**	**Specificity**	**Precision**	**Accuracy**	***F***	**Youden**	**Matthews**
BCI	0.606	0.534	0.980	0.941	0.941	**0.960**	**0.960**	**0.921**	**0.9208**
Daylight	0.510	0.225	1.000	0.882	0.891	0.940	0.942	0.882	0.8866
ECFP4	0.490	0.406	0.980	0.922	0.923	0.950	0.951	0.901	0.9017
ECFC4	0.364	0.415	0.980	0.882	0.889	0.930	0.932	0.862	0.8645
MDL	0.650	0.487	0.939	0.882	0.885	0.910	0.911	0.821	0.8216
Unity	0.639	0.537	0.938	0.961	**0.957**	0.950	0.947	0.898	0.8990

*t*_*ROC*_ is the similarity threshold that gives the best level of performance, where this is that similarity value which maximises the values of the precision, the accuracy, the *F* index, the Youden index and the Matthews coefficient whilst maintaining acceptable values of the sensitivity and specificity. The largest values of these last five variables are bold-faced in the table.

### Use of an external test-set

As described in Experimental methods, a test-set of 100 molecule-pairs was created using data from previous applications to the CHMP for orphan-drug registration. A comparison of the characteristics of these test-set molecules with those in the training-set can be found in Table 3. The Tanimoto similarities were computed for each test-set molecule-pair using each of the six fingerprints, and similarity or non-similarity predicted using the *t*_*LR*_ and *t*_*ROC*_ thresholds developed from the training-set. The results obtained are shown in Table 4, where it will be seen that in all cases the choice of threshold has little or no difference in predictive ability and, importantly, that all but the MDL and ECFC4 fingerprints performed extremely well. The MDL performance is in line with that observed in Table 2 (where it was the worst overall performer) but that for ECFC4 is notably poor: it was the second-worst performer in Table 2 but its performance here is far inferior to that of all of the other fingerprints. Inspection of the 29 molecule-pairs where it failed provided no obvious reason for the incorrect predictions; indeed, given that these test-set molecules are rather larger than those in the training-set this fingerprint might have been expected to do particularly well as it takes account of how frequently each fragment substructure occurs, rather than just its presence or absence as in the other fingerprint-types.

**Table 3:** Characteristics of the 163 training-set and 51 test-set molecules

	**Training-set**	**Test-set**
Molecular weight	301 (100–500)	392 (150–1950)
Number of carbons	16 (5–26)	22 (0–86)
Number of heteroatoms	5 (1–11)	9 (3–52)
Number of rings	2 (0–5)	3 (0–11)
Number of aromatic rings	2 (0–4)	1 (0–3)
Number of stereocentres	1 (0–9)	1 (0–15)

Each element of the table lists the median value, together with the corresponding range in brackets.

**Table 4:** **Numbers of test-set molecule-pairs predicted correctly using *t***_***LR ***_**and *t***_***ROC***_

**Fingerprint**	***t***_***LR***_	***t***_***ROC***_
BCI	97	97
Daylight	97	98
ECFP4	96	97
ECFC4	71	71
MDL	92	92
Unity	97	97
Consensus	98	98

A simple consensus approach was then used to see if further improvements could be made. A molecule-pair was classified as similar or non-similar by each of the fingerprints individually, and then the final classification was similar if three or more of the individual classifications were similar. This consensus result forms the bottom row of Table 4.

## Conclusions

In this paper, we have described how fingerprint-based measures of similarity can be used to assess the structural novelty of molecules that are being submitted for consideration as new medicines for rare diseases. Such measures are well established, dating back to at least the mid-1970s [19], for applications such as property prediction, cluster analysis and virtual screening. A characteristic of most of these studies is that they have focused on the identification of molecules that are similar to each other; in similarity-based virtual screening, for example, the aim is to identify those previously untested database structures that are most similar to a bioactive reference structure [20,21], whilst removing the large numbers of low-similarity database structures from further consideration. In the current application, conversely, dissimilarity is of at least as much importance as is similarity; indeed, it is arguably of greater importance for a company applying for orphan drug authorisation. The other chemoinformatics application where dissimilarity is important is molecular diversity analysis; however the identification of sets of mutually dissimilar molecules is very different from the need to determine whether two molecules are considered to be significantly different as is required for the current application.

The results obtained here demonstrate clearly that simple, 2D fingerprint representations provide measures of structural similarity that mimic closely the judgments of experts, using both training-set molecule-pairs extracted from DrugBank and test-set molecule-pairs typical of the work of the CHMP. This is so despite the fact that the two sets of molecules are rather different in character (as demonstrated by the figures in Table 3). The BCI fingerprints performed best overall on the training-set while the BCI, Daylight, ECFP4 and Unity fingerprints showed comparable, high levels of predictive performance on the test-set. The BCI fingerprints would hence seem to be an appropriate choice for future studies in this area. They encode six different classes of chemical substructure: augmented atoms, atom/bond sequences, atom pairs, and three types of ring feature. The atom- and bond-types can be generalized if required and an algorithmic procedure is used to select the required number of substructures (1052 in the present case) whilst ensuring that they satisfy user-specified criteria relating to minimum, maximum and co-occurrence frequencies.

There are, of course, similarity measures other than those studied to date that could be used for the study of orphan drug similarity, e.g., a measure that takes account of 3D structural information or that uses a similarity coefficient other than the Tanimoto coefficient. Other possible areas of study include the use of multiple similarity measures in the logistic regression model, accounting for individual judgements instead of using the majority decision, or the use of more sophisticated data fusion methods [22] than the simple consensus approach considered thus far.

In conclusion, we must emphasise that we are not suggesting that a computational procedure could be used as an alternative to, let alone a replacement for, the current processes used to evaluate applications for orphan drug authorisations. However, the approach described here could form a useful, quantitative input to those evaluations by providing a tool to assess molecular structural similarity by interested parties. Assume that a new molecule *M* is being submitted for orphan drug authorisation, and that there is already an existing drug *D* for this indication. The similarity between *M* and *D* is computed, e.g. using one of the fingerprint-types that performed well in the experiments above, and then the corresponding regression equation used to give the probability that the two molecules would be considered a similar molecule-pair, based upon experts’ previous similarity assessments. This probability would then be one of the multiple factors that are considered when deciding upon the similarity or otherwise between *M* and *D*[23].

## Experimental methods

In the evaluation of similarity in the context of orphan drug evaluation, the CHMP needs to decide whether or not an active compound for which a new medicine, or an extension of an existing marketing authorisation (change of indication or line extension), is being sought is similar to an existing orphan drug that has already been authorised. The decision is made on the basis of the votes of a panel of experts drawn from each of the member states of the EU. Focussing just on the similarity criterion, the experts are required to make a binary decision: is the new molecule similar to, or different from, the existing orphan drug(s) for the rare disease of interest? The experimental set-up that we have created seeks to mimic this situation, with a panel of experts being asked to make judgements on the similarity or otherwise of a carefully chosen training-set of molecule-pairs, and then a comparison being made between these human decisions and the outputs from computer-based similarity calculations. In this section, we describe the following components of our experimental procedure: the training-set of molecule-pairs on which the similarity assessments were made; the panel of experts who made these assessments; measuring the effectiveness of the automated assessments; and, finally, a second, independent test-set of molecule-pairs that was used to assess the predictive performance of the models developed from the training-set.

### The training-set

The training-set database contains 100 pairs of bioactive molecules selected from DrugBank 3.0 (at http://www.drugbank.ca/), a bioinformatics and chemoinformatics resource that contains a wealth of detailed information on over six thousand drug molecules and their associated biological targets [24]. The file was filtered to identify 1068 molecules that contained at least one carbon atom and that had not more than ten hydrogen bond accepters, not more than five hydrogen bond donors, a molecular mass not greater than 500 Daltons, and an octanol-water partition coefficient not greater than 5. The similarity between each distinct pair of these drug-like molecules was computed using ECFP4 fingerprints [25] and the Tanimoto coefficient [26], and then 100 pairs of molecules chosen so as to cover as wide and as equal a spread of Tanimoto values as possible, with the observed similarity values ranging from 0.116 to 1.000. The molecule-pairs contained a total of 163 distinct molecules, these representing 42 different pharmacological classes including antibiotics, beta-adrenergic antagonists, benzodiazepines and anti-hypertensives *inter alia*.

### The experts

With the permission of the EMA, one of us (PF) gave presentations to several EMA committees and working parties responsible for the evaluation and the quality of medicines. The attendees at these meetings who had a background in quality and experience in assessing orphan drugs were invited to participate in the project by providing similarity judgments on the 100 pairs of DrugBank molecules. Similar invitations were sent to appropriate individuals on an EMA email list of European experts with a background in the quality of medicines, and to contact points in the regulatory authorities in the USA, Japan and Taiwan (the Food and Drug Administration, the Pharmaceutical and Medical Devices Agency, and the Food and Drug Administration of Taiwan, respectively). Participants were sent the 100 pairs of 2D structure diagrams and for each molecule-pair asked to state whether (Yes) or not (No) the two molecules should be regarded as being structurally similar. A total of 143 completed responses (128 from within the EU) was obtained and these were then used to compute the fractions of Yes and No responses for each of the pairs of molecules. The structure diagrams and SMILES descriptions for the 100 molecule-pairs and the percentages of Yes and No responses for each such pair are listed in Additional file 1: Table S1.

The decisions of the CHMP are decided on the basis of majority voting, and it was hence decided that the molecule-pairs in the sample where more than 50% of the responses were Yes should be considered as similar, which we shall refer to as a *similar molecule-pair*. If this was not the case then the two molecules were judged to be a *non-similar molecule-pair*. This resulted in 49 similar molecule-pairs and 51 non-similar molecules-pairs; then, once each of the molecule-pairs had been categorised in this way, the expert judgments were used to assess the categorisation ability of similarity measures based on 2D fingerprints.

### Measurement of effectiveness

Many different types of structural representation can be used to compute inter-molecular structural similarities. The similarities here were computed with 2D fingerprints, which are widely used for this purpose since they are both simple to compute and effective in operation [26,27]. The following types of fingerprint were generated to represent the molecules in each of the pairs: BCI (1052), Daylight (2048), ECFC4 (1024), ECFP4 (1024), MDL (166) and Unity (988), where the number in brackets is the number of elements in the fingerprint. Brief descriptions of all these types of fingerprint are provided by Gardiner *et al*. [28]. The Tanimoto coefficient was used to compute the similarity between the fingerprints for each of the molecules comprising a molecule-pair, using each type of fingerprint in turn. In addition, 23 computed molecular properties (such as molecular weight, logP, pKa, molar refractivity, PSA, numbers of rotatable bonds and stereocentres etc.) were computed using Pipeline Pilot to provide an additional type of structure representation. However, the results obtained using this representation were uniformly inferior to those obtained using the various 2D fingerprints, and the results and discussion hence consider only the fingerprint-based similarity data.

The ability of each type of fingerprint to predict similarity between molecules was determined using logistic regression, receiver operating characteristic (ROC) curves, and a range of measures of predictive success taken from the information retrieval and machine learning literatures. For the logistic regression analysis, let *p* be the probability that the panel will conclude that two molecules do indeed comprise a similar molecule-pair given a computed similarity *x*. Then logistic regression yields an equation of the form

$$\mathrm{logit}\left(p\right)=\ln\left(\frac{p}{1-p}\right)=\beta_{0}+\beta_{1}x$$

that describes a linear relationship between the similarity and the logarithm of the odds that the molecules comprise a similar molecule-pair [29]. The performance of the model can be assessed by observing the differences between the sets of observed and predicted values: this was done here using Nagelkerke’s *R*^2^ statistic, which takes values between zero and unity (denoting a very poor fit and a perfect fit, respectively). Also, Hosmer-Lemenshow goodness-of-fit tests were used to assess the assumptions of the model (i.e., linearity at the log scale and additivity). Once the logistic regression equation for a fingerprint had been generated, it was used to compute the threshold similarity, *t*_*LR*_, such that the two molecules comprising a pair are predicted to be similar (‘Yes’) if their computed similarity is ≥ *t*_*LR*_ (corresponding to a probability greater than or equal to 0.5 of being similar according to the logistic regression model) or predicted to be not similar (‘No’) if < *t*_*LR*_ (corresponding to a probability lower than 0.5 of being similar according to the logistic regression model).

The chosen cut-off probability of 0.5 may not necessarily be the best value to discriminate between similar and non-similar pairs. ROC curves built from the predicted probabilities of the logistic model provide an alternative way of identifying an appropriate threshold similarity, here called *t*_*ROC*_, for deciding that two molecules represent a similar molecule-pair. Assume that a particular similarity measure has been chosen. Then let *t* denote some threshold similarity for that measure such that the two molecules comprising a pair are predicted to be similar if their computed similarity is ≥ *t* and not similar if < *t* (in the same way as defined for *t*_*LR*_). These predictions can then be compared with the expert judgements in terms of true positives (*TP*), true negatives (*TN*), false positives (*FP*) and false negatives (*FN*) where, e.g., *TP* is the number of cases where the majority of the experts judged two molecules to form a similar molecule-pair and where those two molecules had a computed similarity ≥ *t*. Knowing these four values it is possible to compute the specificity,

$$\frac{\mathrm{TN}}{\mathrm{TN}+\mathrm{FP}},$$

and the sensitivity,

$$\frac{\mathrm{TP}}{\mathrm{TP}+\mathrm{FN}},$$

for that value of *t*, and hence to plot the ROC curve obtained by systematically varying *t*. The area under the curve (hereafter AUC) then provides a measure of the extent to which the computed similarity measure mirrors the expert judgements, with values close to unity indicating the highest levels of agreement. In addition to specificity and sensitivity, the following performance statistics were computed: the precision

$$\frac{\mathrm{TP}}{\mathrm{TP}+\mathrm{FP}};$$

the accuracy

$$\frac{\mathrm{TP}+\mathrm{TN}}{\mathrm{TP}+\mathrm{TN}+\mathrm{FP}+\mathrm{FN}};$$

the *F* index

$$\frac{2\times \mathrm{TP}}{2\times \mathrm{TP}+\mathrm{FP}+\mathrm{FN}};$$

the Youden index

TP×TP−FP×FN;

and Matthew’s correlation coefficient

$$\frac{\mathrm{TP}\times \mathrm{TN}-\mathrm{FP}\times \mathrm{FN}}{\sqrt{\left(\mathrm{TP}+\mathrm{FP}\right)\left(\mathrm{TP}+\mathrm{FN}\right)\left(\mathrm{TN}+\mathrm{FP}\right)\left(\mathrm{TN}+\mathrm{FN}\right)}},$$

These performance statistics were computed as the value of *t* was systematically varied, so as to determine *t*_*ROC*_, i.e., the threshold similarity that resulted in the best overall predictive performance. The best level of performance was taken to be that threshold similarity which resulted in the maximum values for the precision, the accuracy, the *F* index, the Youden index and the Matthews coefficient whilst maintaining acceptable values of the sensitivity and specificity.

### The test-set

The predictive power of the models derived from the similarity data for the 100 DrugBank molecule-pairs could have been assessed by means of cross validation experiments using that training-set, as is often done in SAR and QSAR studies. It is generally considered better, however, to use a distinct test-set that has not been involved in the training [30,31], and this was accomplished here using data kindly provided by the CHMP that typifies their regular work-load. Specifically, the test-set contained 100 molecule-pairs in which one molecule was an existing orphan drug for some specific rare disease and the other was a molecule that had been submitted to the CHMP for consideration for orphan drug status for that disease.

It should be noted that the test-set differs from the training-set in two principal ways. First, of the 100 molecule-pairs provided by the CHMP, 89 of them had been judged to be non-similar pairs with only 11 judged to be similar pairs, whereas the test-set contained near-equal numbers of the two types of molecule-pair. This is not unexpected given that companies are unlikely to submit for consideration molecules that are obviously closely related to existing orphan drugs for some disease. Second, the natures of the molecules involved. It is not possible to provide structural data analogous to that presented in Additional file 1: Table S1 given the highly confidential nature of the application process. However, some broad characteristics of the test-set are as follows. There were 51 distinct molecules in the test-set, since many of the molecule-pairs resulted from the comparison of an existing orphan drug with several different molecules that had sought authorisation for the same rare disease. Just five pharmacological classes were represented: there were two immunosuppressants, two respiratory system compounds, three antimicrobials, ten pulmonary arterial hypertension compounds, and no less than 34 antineoplastic compounds (reflecting the fact that much current orphan drug research focuses on therapies for rare types of cancer [7]). The compounds were notably larger than those in the test-set as demonstrated in Table 3, with 27.5% of them not being Lipinski-compliant.

## Competing interests

The authors declare that they have no competing interests. The views expressed in this article are the personal views of the authors and may not be understood or quoted as being made on behalf of or reflecting the position of the European Medicines Agency or any of its committees or working parties.

## Authors’ contributions

PF carried out the experiments with assistance from JH. NP carried out the statistical analyses. PW conceived the study, participated in its design and coordination, and drafted the manuscript with assistance from PF, JH and NP. All authors read and approved the final manuscript.

## Supplementary Material

Additional file 1: Table S1 The 100 molecules-pairs from DrugBank 3.0 that comprised the training-set on which the 143 experts provided Yes/No decisions. Click here for file
